# Study of the brainstem auditory evoked potential with speech stimulus in the pediatric population with and without oral language disorders: a systematic review^☆^

**DOI:** 10.1016/j.bjorl.2020.05.025

**Published:** 2020-07-18

**Authors:** Jéssica Dayane da Silva, Lilian Ferreira Muniz, Mariana de Carvalho Leal Gouveia, Laís Cristine Delgado da Hora

**Affiliations:** a Universidade Federal de Pernambuco (UFPE), Programa de Pós-Graduação em Saúde da Comunicação Humana, Recife, PE, Brazil; b Universidade Federal de Pernambuco (UFPE), Departamento de Fonoaudiologia, Recife, PE, Brazil; c Universidade Federal de Pernambuco, Departamento de Cirurgia, Recife, PE, Brazil

**Keywords:** Auditory evoked potentials, Speech perception, Child, Adolescents, Systematic review, Potenciais evocados auditivos, Percepção da fala, Criança, Adolescentes, Revisão sistemática

## Abstract

**Introduction:**

The brainstem auditory evoked potential with speech stimulus, BAEP-speech, has been applied to observe how speech sounds are manifested in the brainstem. This tool can be used in children to assess central auditory processing, allowing preventive and early interventions.

**Objective:**

To assess the results found in the brainstem auditory evoked potential with speech stimulus in the pediatric population with and without oral language disorders, through a systematic literature review.

**Methods:**

The search was carried out in the scientific databases Portal BVS, Pubmed, Lilacs, Medline, Scielo and Web of Science, OpenGrey.eu, DissOnline, OpenDoar, OAIster and The New York Academy of Medicine. A systematic literature review was performed using the descriptors: auditory evoked potentials, children and their synonyms, combined by the Boolean operators AND and OR. The search filter “age: child” was used. The studies were independently read by peers and, in case of disagreement regarding the inclusion of studies, a third researcher was consulted. Original case-control articles that performed BAEP-speech without competitive noise, carried out in the pediatric population without and with oral language disorders, were included.

**Result:**

14 articles published between 2008 and 2019 were included in this review. Methodological variability was observed in the exam, with the syllable / da / being the most frequently used as the stimulus. When performing the average of the groups, it was observed that the population with specific language disorders showed greater latency delays in the sustained portion, lower amplitude values and VA complex slope. The group with phonological disorders had higher values in the transient portion of the responses.

**Conclusion:**

Children with language disorders of different etiologies have different patterns of BAEP-speech responses when compared to children with typical development.

## Resumo

**Introdução:**

O potencial evocado auditivo de tronco encefálico com estímulo de fala (PEATE-fala) tem sido aplicado para observar como os sons da fala se manifestam no tronco encefálico. Essa ferramenta pode ser usada em crianças na avaliação do processamento auditivo central, permite intervenções preventivas e precoces.

**Objetivo:**

Conhecer os resultados encontrados no potencial evocado auditivo de tronco encefálico com estímulo de fala na população pediátrica com e sem transtornos de linguagem oral, por meio de revisão sistemática da literatura.

**Método:**

As buscas foram feitas nas bases de dados científicos Portal BVS, Pubmed, Lilacs, Medline, Scielo e Web of Science, OpenGrey.eu, DissOnline, OpenDoar, OAIster e *The New York Academy of Medicine*. Foi feita revisão sistemática da literatura, com os descritores *auditory evoked potentials*, *children* e seus sinônimos, combinados pelos operadores booleanos AND e OR. Foi usado o filtro de pesquisa “idade: criança”. A leitura dos estudos foi feita por pares de forma independente e em caso de discordância na inclusão de estudos um terceiro pesquisador foi consultado. Foram incluídos artigos originais do tipo caso-controle que fizeram o PEATE-fala sem ruído competitivo na população pediátrica sem e com transtornos de linguagem oral.

**Resultado:**

Foram incluídos 14 artigos publicados entre 2008 e 2019 na presente revisão. Foi observada variabilidade metodológica na realização do exame, a sílaba /da/ foi a mais usada para estimulação. Ao se fazerem médias dos grupos, observou-se que a população com distúrbio específico de linguagem apresentou maiores atrasos de latência na porção sustentada, menores valores de amplitude e *slope* do complexo VA. O grupo com transtorno fonológico obteve maiores valores na porção transiente das respostas.

**Conclusão:**

Crianças com alterações de linguagem de diferentes etiologias apresentam padrões de respostas do PEATE-fala distintos quando comparadas às crianças com desenvolvimento típico.

## Introduction

The integrity of the peripheral and central auditory system is essential for the oral language acquisition process. The understanding of the acoustic elements and their phonetic representations as language formants comprise one of the means of speech development. Receptive hearing impairment can hinder the coding of acoustic speech signals, affecting the child's communication. Therefore, analysis of the auditory pathway function must be performed in case of difficulties in learning^1^ spoken language.

The brainstem auditory evoked potential (BAEP) is widely used in clinical practice to objectively assess the integrity of the auditory pathways to the brainstem, in addition to estimating the electrophysiological hearing threshold. This examination can be performed with the click, tone pip, or tone burst stimulation, or speech.^2^, ^3^ However, brief signals have a simple acoustic pattern that is different from the sounds found in the environment, such as verbal sounds, making them limited in the assessment of brainstem behavior, especially when considering the speech sound processing in these structures.^4^

The brainstem auditory evoked potential with speech stimulus (BAEP-speech) can be indicated to understand how the auditory pathway processes verbal sounds, as it allows observing the acoustic properties of the speech formants that appear preserved in the brainstem responses,^5^ different from the aforementioned stimuli.

Therefore, it provides information on how a syllable is encoded by the auditory system, constituting a method of neural capacity analysis to assess changes in the components of time and frequency present in the acoustic stimulus.^4^, ^6^, ^7^

Thus, the BAEP-speech has been shown to be an important tool that can help in the assessment of central auditory processing, allowing a preventive intervention, even without the results of a behavioral assessment, which is difficult to perform in some children.^5^, ^8^

Currently, the great challenge has been to establish normality standards for this procedure. Therefore, it is necessary to study the use of auditory evoked potential with speech stimulus to observe the central auditory processing of speech sounds in the pediatric population with and without oral language disorders, observing response patterns described in the literature that allow and facilitate the safer use of the procedure in clinical practice.

The aim of the present study was to determine the results found in the brainstem auditory evoked potential with speech stimulus in the pediatric population with and without oral language disorders, through a systematic literature review.

## Methods

The literature review was approved on the PROSPERO platform under registration number CRD42019119322.

The following guiding question was used to carry out this study: Are there any differences in the results found in the brainstem auditory evoked potential with speech stimulus in the pediatric population with and without oral language disorders?

### Search strategy

The term “pediatric population” includes individuals from birth to eighteen years of age, and the Medical Subject Headings descriptors “pediatrics”, “child” and “adolescent” were used as basis for its creation and delimitation of the age group.

The Latin American and Caribbean Health Sciences (Lilacs), Medical Literature Analysis and Retrieval System Online (Medline) via PubMed and Scientific Electronic Library Online (SciELO), Web of Science and the Virtual Library of Saúde (BVS Portal) databases were searched, as well as the gray literature bases OpenGrey.eu, DissOnline, OpenDoar, OAIster and The New York Academy of Medicine. There were no language or publication date restrictions. The following search key was used: auditory evoked potential OR evoked potential, auditory OR potentials, auditory evoked OR auditory evoked response OR auditory evoked responses OR evoked response, auditory OR evoked responses, auditory OR auditory evoked potentials AND children OR child. The “Age: Child” search filter was used. The database search was carried out in March and April of 2020.

### Eligibility criteria

The review included only original case-control articles that performed BAEP-speech without competitive noise in the pediatric population with and without oral language disorders. Only case-control articles were included considering the need to compare the population with oral language disorders to those with typical development, being essential to perform the evaluation of similar age groups and using the same examination parameters. Literature reviews, studies without a control group, repeated articles in different databases, studies in non-human beings, as well as studies carried out in individuals with hearing loss, neurological syndromes and brainstem alterations were excluded.

### Study selection

The search was carried out by two independent reviewers, and, in the absence of agreement, the study was evaluated by a third researcher for final decision making.

The first phase of article selection included the reading of titles and abstracts of all identified studies. After excluding the ones that did not meet the objective of the current study and did not meet the eligibility criteria, duplicate articles were excluded. The remaining ones were then read in full, which led to the exclusion of studies that did not meet the review proposal.

### Data extraction and quality assessment

The data extracted from the study results were: names of the authors, year of publication, country, sample size, age group of the studied group, type of oral language alteration, examination stimulation parameters, in addition to the mean slope values, amplitude, latencies, areas and main conclusions made available by the studies.

The Newcastle-Ottawa scale adapted for cross-sectional observational studies was used to assess study quality, which was independently evaluated by two researchers based on the following items: 1) Sample representativeness; 2) Sample size; 3) Management of non-responses; 4) Calculation of exposure (risk factor); 5) Comparability of subjects in different groups of results based on the design or analysis (control of confounding factors); 6) Evaluation of results; 7) Statistical test.

## Results

**Figure 1:**
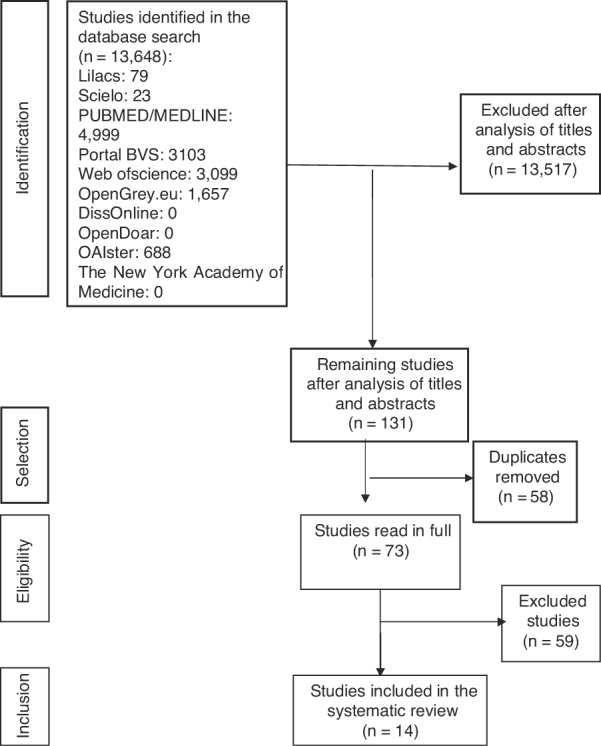
Flow diagram of article selection.

A total of 13,648 titles were identified in the initial search. After the reading of the titles and abstracts, 131 studies remained, of which 58 duplicates were removed, with 73 of them being read in full. After reading the full texts, 59 studies were excluded, due to the following reasons: Adult population (15), brain trauma (1), brainstem injury (1), studies that did not indicate the age group of the population (2), BAEP-speech was performed with competitive noise (4), materials from scientific events (5), studies without a control group (10) and absence of oral language disorder in at least one studied group (21). Finally, 14 studies were selected for the current review (Fig. 1).

Table 1 shows the data obtained after reading the articles, according to what was pre-established for data extraction. Of the included studies, five aimed at assessing individuals with Autism Spectrum Disorders (ASD), five assessed participants with Specific Language Disorders (SLD), two observed children with Phonological Disorders (PD), one studied persistent developmental stuttering, and another assessed individuals with Attention-Deficit/Hyperactivity Disorder (ADHD).

**Table 1:** Study characteristics, stimulation parameters and mean values of slope, amplitude, latency and BAEP-speech area.

Language alteration	Author/Year	Country	Sample size (participants)	Age	Stimulation parameters	Slope values (mv/ms)	Amplitude values de (mv)	Latency value (ms)	Area values (mv × ms)	Main conclusions
PD	Ferreira, L. et. al., 2019	Brazil	60	5 − 8,11 years	Syllable / of /, 40 ms, right ear (RE), intensity of 80 dB nHL, impedance up to 3kΩ, presentation rate of 10.9 s, window of 80 − 100 ms, bandpass filter 100 − 2000 Hz, two scans of 3000 stimuli.	Group I: 0.55	x	Group 1:	Grupo I: 0,56 Grupo II: 0,77	Children with PD show disorganization in the neural coding of complex sounds, due to changes in the BAEP-speech responses. This could compromise the development of language skills, which can reflect on communication.
						Group II: 0.38		V: 6.79		
								A: 7.84		
								C: 17.43		
								D: 23.07		
								E: 32.07		
								F: 39.96 O: 47.97		
								Group 2:		
								V: 7.18		
								A: 8.66 C: 18.32		
								D: 23.23		
								E: 32.47		
								F: 41.73		
								O: 49.06		
	Gonçalves, I. C., et. al., 2011	Brazil	36	7 − 11 years	Syllable [da], 40 ms, RE, intensity of 80 dBA, impedance of up to 5kΩ, presentation rate of 11.1 stimuli / s, band-pass filter 100 − 2000 Hz, 2000 stimuli.	VA complex: Group 1: 0.36 Group 2: 0.43	Group 1: V: 0,32 A: −0,31 C: −0,34 F: 0,33 VA Complex: 0.69 Group 2: V: 0.30 A: −0.35 C: -0.35 F: 0.37 VA Complex: 0.67	Group 1: V: 7,41 A: 9,39 C: 19,42 F: 40,10 VA Complex: 1.97 Group 2: V: 8.58 A: 10.32 C: 18.76 F: 40.00 VA Complex: 1.74	x	They indicate that the initial stages of processing an acoustic stimulus in the auditory pathway are not similar in children with typical development and with PD. They suggest that the abnormal encoding of speech sounds may be a biological marker of PD.
TEA	Chen, J., et al., 2019	China	35	3 − 6 years	Syllable [da], 40 ms, RE, intensity of 80 dB SPL, impedance of up to 5kΩ, presentation rate of 10.9 stimuli / s, three scans of 3000 stimuli.	x	Mean values in the first and second evaluations. respectively:	Mean values in the first and second evaluations. respectively:	x	Children with ASD show subcortical processing that is still immature when compared to those with DD.
							Group 1:	Group 1:		
							V: 0.10/ 0.11; A: −0.20/−0.23 C: −0.14/	V: 6.66/6.54 A: 7.58/7.51		
							−0.11	C: 18.37/ 18.20		
							D: −0.14/	D: 22.58/ 22.39		
							−0.18	E: 31.30/ 31.32		
							E: −0.22/	F: 39.60/ 39.32 O: 48.04/48.07 Group 2:		
							−0.23	V: 6.90/6.73 A: 7.83/7.86 C: 18.47/18.43		
							F: −0.27/	D: 22.55/ 22.99		
							−0.19	E: 31.13/ 31.08 F:39.74/39.58 O: 48.29/48.17		
							O: −0.21/ −0.18 Group 2:			
							V: 0.09/ 0.11 A: −0.18/−0.22 C: −0.04/			
							−0.07			
							D: −0.19/			
							−0.21			
							E: −0.16/ −0.18			
							F: −0.18/			
							−0.18			
							O: −0.09/			
							−0.14			
	Ramezani, M., et. al., 2018	Iran	56	Mean ages: 14.36 − 14.99	Syllable [da], 40 ms, RE, 80 dB SPL intensity, impedance up to 5kΩ, intermittent polarity, presentation rate of 10.9 stimuli / s, band-pass filter 100−2000 Hz, two scans of 3000 stimuli.	x	x	Group 1: V: 6.32 A: 7.18 C: 18.03 D: 22.10 E: 30.74 F: 39.12 O: 47.68 Group 2: V: 6.89 A: 7.82 C: 20.01 D: 24.60 E: 33.35 F: 41.12 O: 49.58	x	There are losses in the synchronization of neural activity in individuals with ASD, indicating a dysfunction in the speech stimulus processing in the brainstem.
	Otto-Meyer, S., et. al., 2018	USA	24	7 − 13 years	Stimulus / d /, 40 ms, RE, 80 dB SPL intensity, impedance up to 5kΩ, alternating polarity, 10.9 ms presentation rate, 75 ms window, 100 Hz high-pass filter and 2000 Hz low-pass filter, two scans of 2000 stimuli. Syllable [ya] going up and [ya] going down, 230 ms, RE, 60 dB SPL intensity, alternating polarity, 80 − 1000 Hz band-pass filter, 4800 stimuli.	x	x	x	x	Children with high-performance ASD have less stable BAEP-speech than children with typical development, persisting through multiple stimuli. The effects can go beyond the auditory system, given the integrated nature of the BAEP-speech generators.
	Russo, N., et. al., 2009	USA	39	7 − 13 years	Syllable [da], 40 ms, RE, 80 dB SPL intensity, alternating polarity, impedance up to 5kΩ, presentation rate of 10.9 stimuli/s, 75 ms window, 100 − 2000HZ band-pass filter, three scans of 2000 stimuli.	x	x	Group 1: V: 6.54 A: 7.48 D: 22.38 F: 39.25 Group 2: V: 6.73 A: 7.85 D: 22.77 F: 39.54	x	There is a reduction in neural synchrony and phase block in speech stimulation.
	Russo, N. M., et. al., 2008	USA	42	7 − 13 years	Syllable [ya], 230 ms, RE, 60 dB SPL intensity, impedance up to 5kΩ, 50 ms window, alternating polarity, 80 − 1000 Hz band-pass filter, two scans of 1200 stimuli per polarity.	x	x	X	x	Some children with ASD show marked deficiencies in pitch tracking. In addition, they indicate a reduction in the phase block.
Stuttering	Mozaffarilegha, M.; Adeli, H., 2018.	Iran	29	15 − 33 years	Syllable [da], 40 ms, RE, 80 dB SPL intensity, alternating polarity, 85.33 ms window, 6000 stimuli.	x	x	X	x	Promising capacity of the graphic index complexity (GIC) to assess abnormal activation of the brainstem level in the study group. They associate the visibility of BAEP graphs with mechanisms associated with long-term memory of the auditory system dynamics at the brainstem level.
DEL	Gabr, T. A.; Darwish, M. E., 2015	Egypt	40	3 − 7 years	Syllable [da], 206 ms, both ears, 70 dB nHL intensity, alternating polarity, presentation rate of 11.1 stimuli / s, 75 ms window, band-pass filter 50 − 1000HZ, three scans of 1024 stimuli.	VA Complex: Group 1: 0.426 Group 2: 0.195	Group 1:	Group 1:	Group 1: 1.65 Group 2: 2.05	Children with SLD have abnormal coding for specific acoustic characteristics, reduced neural synchrony for transient changes in speech and impaired neural coding for the duration of the stimulus. Manifesting as abnormal BAEP-speech.
							V: 0.468	V: 6.60		
							A: 0.319	A: 8.73 C: 14.04		
							C: 0.446	D: 21.0		
							D: 0.513	E: 29.1		
							E: 0.818	F: 37.9		
							F: 0.95	O: 47.00 Group 2:		
							O: 0.765 VA Complex: 0.787 Group 2:	V: 7.95		
							V: 0.13	A: 12.1		
							A: 0.86	C: 21.0		
							C: 0.159	D: 29.7		
							D: 0.111	E: 38.6		
							E: 0.199	F: 47.3		
							F: 0.188	O: 55.8		
							O: 0.31 VA Complex: 0.334			
	Rocha-Muniz, C. N., Befi-Lopes, D. M., & Schochat, E., 2014	Brazil	75	6 − 12 years	Syllable [da], 40 ms, RE, impedance up to 5kΩ, alternating polarity, 74.67 ms window, band-pass filter 100 − 2000 Hz, three scans of 1000 stimuli.	x	x	Group 1: V: 6.32 A: 7.87 C: 17.57 D: 22.83 E: 30.64 F: 39.37 O:48.01 Group 2: V: 6.78 A: 8.61 C: 18.76 D: 23.72 E: 31.79 F: 41.14 O: 49.27	x	BAEP-speech can be used to diagnose language impairments, considering other factors and the presence of risks. Suggested use of the Brazilian procedure in English speakers, since the results obtained for the syllable [da] are similar for speakers of both languages.
	Rocha-Muniz, N. C.; Befi-Lopes, M. D.; Schochat, E., 2012	Brazil	57	6 − 12 years	Syllable [da], 40 ms, RE, impedance up to 5kΩ, alternating polarity, 74.67 ms window, band-pass filter 100 − 2000 Hz, three scans of 1000 stimuli.	x	x	Group 1: V: 6.31 A: 7.35 C: 17.54 D: 22.83 E: 30.79 F: 39.17 O: 48.13 Group 2: V: 6.78 A: 8.75 C: 18.89 D: 23.80 E: 32.01 F: 41.48 O: 49.39	x	The group with SLD showed alterations for temporal measures and frequency coding measures. Abnormal BAEP-s should manifest as difficulties in speech perception. The group with SLD showed worse responses than the others.
	Filippini, R.; Befi-Lopes, M. D.; Schochat E., 2012	Brazil	Three groups: 1: DD 2: SLD (formal auditory training and speech therapy) 3: SLD (speech therapy only)	7 − 13 years	Syllable [da], 40 ms, RE, 80 dB nHL, alternating polarity, presentation rate of 10.9 stimuli / s, 74.67 ms window, band-pass filter of 100 − 2000 Hz, three scans of 1000 stimuli.	VA complex, before and after auditory training, respectively: Group 1: 0.45 / 0.37 Group 2: 0.33 / 0.32 Group 3: 0.35 / 0.38	VA complex, before and after auditory training, respectively: Group 1: 0.41 / 0.34 Group 2: 0.29 / 0.29 Group 3: 0.35 / 0.35	Means before and after auditory training, respectively. Group 1: V: 6.53/6.63 A: 7.45/7.60 C: 18.43/18.21 D: 22.22/22.45 E: 31.04/31.16 F: 39.41/39.33 O:48.10/47.94 VA Complex: 0.92/0.92 Group 2: V: 6.64/6.67 A: 7.51/7.58 C: 18.74/18.56 D: 22.51/22.22 E: 31.45/30.72 F: 39.59/39.23 O: 47.88/47.93 VA Complex: 0.88/0.91 Group 3: V: 6.59/6.59 A: 7.63/7.48 C: 18.89/18.58 D: 22.39/22.14 E: 30.80/31.02 F: 39.27/39.37 O: 48.13/47.95 VA Complex: 1.02/0.90		Deficits in auditory processing in behavioral and electrophysiological assessments in the SLD groups, similar to those observed in participants with auditory processing disorder. Additionally, the benefits of formal auditory training and formal auditory training associated with speech therapy were observed in behavioral and electrophysiological responses.
	Basu, M.; Krishnan, A.; Weber-Fox, C., 2009	USA	20 participants. Two groups: I: SLD II: DD	4 − 11 years	Syllables [ba] and [de], 150 ms, RE, intensity of 50 dB nHL, impedance up to 3kΩ, band-pass filter 100 − 3000 Hz.	x	x	X	x	Children with SLD have a temporal deficit at the brainstem level, sounds with rapid presentations or changes are not adequately coded. The assessed population showed a marked interruption in the neural phase block.
TDAH	Jafari, Z.; Malayeri, S.; Rostami, R., 2015	Iran	84	8 − 12 years	Syllable [da], 40 ms, RE, intensity of 80 dB SPL, impedance up to 5kΩ, presentation rate of 10.9 stimuli / s, band-pass filter 100 − 2000 Hz, two scans of 2000 stimuli.	x	x	Group 1: V: 5.920 A: 6.749 C: 17.366 D: 21.178 E: 30.047 F: 38.690 O: 46.788 Group 2: V: 5.935 A: 7.065 C: 17.565 D: 21.716 E: 30.466 F: 39.04 O: 47.08	x	BAEP-speech indicating delay in temporal coding in children of the study group. Children with ADHD have dysfunction in processing speech stimuli and no speech at the brainstem level.

In the Chart, Group 1 will always represent the control group, while Group 2 will correspond to the study group.

The studies included in the review were produced between the years 2008 and 2019; five of them were produced in Brazil, four in the United States, three in Iran, one in Egypt and one in China. Twelve of these studies used the syllable / da / for the exam, one used the English syllable / of /, another used / ya / and only one of them stimulated with two syllables / ba / and / de /; the predominance of the stimulus / da / can be observed.

In the included articles, one can observe the values of latency^9^, ^10^, ^11^, ^12^, ^13^, ^14^, ^15^, ^16^, ^17^, ^18^ amplitude,^12^, ^16^, ^17^ VA complex slope^10^, ^12^, ^16^, ^17^ and area.^10^, ^12^ Four of the studies^19^, ^20^, ^21^, ^22^ did not show any numerical analysis of the results.

Based on the numerical analyses presented in the assessed studies, a statistical analysis was performed to observe the mean, minimum, maximum and standard deviation values for language pathology and a group for typical development, as shown in Table 2. As the numerical values for latency, amplitude and slope of the BAEP-speech waves were not found in all studies, only those that contained such information were used.

**Table 2:** Numerical analysis of data extracted from the studies included in the review.

	Latencies							Amplitude	Slope
	V	A	C	D	E	F	O	VA Complex	VA Complex
**Phonological disorder**^9^, ^16^									
Mean	7.88	9.49	18.54	x	x	40.86	x	x	0.37
Minimum	7.18	8.66	18.32	x	x	40	x	x	0.36
Maximum	8.58	10.32	18.76	x	x	41.73	x	x	0.38
Standard deviation	0.989	1.173	0.311	x	x	1.223		x	0.014
**SLD**^11^, ^13^, ^14^									
Mean	7.02	9.27	19.38	24.9	33.3	42.29	50.64	0.342	0.322
Minimum	6.59	7.63	18.76	22.39	30.8	39.27	48.13	0.334	0.195
Maximum	7.95	12.1	21	29.7	38.6	47.3	55.8	0.35	0.45
Standard deviation	0.623	1.949	1.078	3.263	3.572	3.473	3.481	0.011	0.18
**TEA**^9^, ^10^, ^17^									
Mean	6.78	7.85	19.22	23.45	32.21	40.08	48.87	x	X
Minimum	6.73	7.82	18.43	22.77	31.08	39.54	48.17	x	X
Maximum	6.89	7.88	20.01	24.6	33.35	41.12	49.58	x	X
Standard deviation	0.092	0.03	1.117	0.999	1.605	0.900	0.997	x	X
**Typical development**^9^, ^10^, ^11^, ^13^, ^14^, ^15^, ^16^, ^17^, ^18^, ^19^									
Mean	6.52	7.75	17.55	22.17	30.71	39.28	46.13	0.59	0.43
Minimum	5.92	6.74	14.04	21	29.1	37.9	40.68	0.41	0.35
Maximum	7.41	9.39	19.42	23.07	32.07	40.1	48.1	0.78	0.55
Standard deviation	0.388	0.773	1.470	0.683	0.876	0.622	3.00	0.266	0.082

There were not sufficient data available to extract the results concerning the VA component area. The articles that evaluated BAEP-speech in individuals with persistent developmental stuttering and ADHD were not included, considering the presence of only one study assessing each of these pathologies, with insufficient data for analysis.

As for the latency, it can be observed that the results regarding all oral language pathologies have higher values when compared to the group with typical development.

The population with PD showed the highest latency values in the V and A waves, when compared to other groups, highlighting the greater difficulty in the transient portion of the stimuli, showing impairment in the perception and encoding of consonants during speech. The group with SLD showed greater delays in the wave latencies of the sustained portion of the stimuli in comparison to the other populations, being indicative of greater impairment in the encoding of vowels with speech stimulus. Individuals with SLD and ASD have a delayed O wave in comparison to children with typical development, showing difficulty in perceiving the end of the stimulus.

As for the amplitude, it was possible to observe only the values for the VA complex of the population with SLD, which was lower when compared to individuals with typical development. This population also showed the lowest value for the slope of the same component, indicating greater temporal difficulty and lower neural discharge in responses to the speech stimulus.

Additionally, it is noteworthy that the group of studies that included SLD showed the largest standard deviations in the latency values in relation to the other groups, indicating great variability in the numerical analyses of the included articles and difficulty in attaining a consensus regarding the findings in this population.

Table 3 describes the results as altered, separated by pathology, of the population with oral language disorders when compared to children with typical development. There is an increase in wave latencies in different pathologies. The article that assessed individuals with persistent developmental stuttering did not carry out analyses regarding this parameter; however, it was the only one that performed a study of graphic representations of BAEP-speech.

**Table 3:** Main changes in BAEP-speech in children with oral language disorders, when compared to the population with typical development.

	Specific language disorder	Autism spectrum disorder	Phonological disorder	Persistent developmental stuttering	Attention deficit hyperactivity disorder
**BAEP-speech alterations**	Increased wave latency;	Increased wave latency;	Increased wave latency; VA complex slope reduction;	Greater complexity of the graphic index;	Increased wave latency;
	Amplitude reduction;	Reduction of the VA complex slope;			Longer duration of the VA complex;
	Reduction of frequency encoding phase block;	Speech pitch encoding impairment;			Reduction of amplitude and slope of the VA complex;
		Phase block reduction;			Extension of the initial phase of the response;
	Changes in wave morphology	Lower representation of early speech;			Lower signal-to-noise ratio;

Moreover, reductions in wave amplitude and VA complex slope are common findings in the population with oral language disorders. Considering that only one article recorded the tracking of frequency coding in speech, in individuals with ASD, the deficiency in pitch coding is pointed out only once.

Study quality was assessed according to the Newcastle-Ottawa Scale adapted for cross-sectional studies; six studies^9^, ^10^, ^11^, ^12^, ^15^, ^20^, ^22^ were classified as very good and seven^13^, ^14^, ^16^, ^17^, ^18^, ^19^, ^21^ were classified as good.

## Discussion

BAEP-speech has shown to be a promising exam, as well as a reliable instrument for the assessment and monitoring of children's hearing, especially those with language disorders.

Due to the presence of different language pathologies identified after the assessment, the studies will be divided into sections, aiming at a better understanding of the results that constitute them, thus allowing a greater understanding of the different findings in the pathologies and comparisons between the authors.

### Specific language disorders

Studies that evaluated children with SLD showed comparisons with different groups. Filippini et al.^16^ performed the BAEP-speech before and after acoustically controlled auditory training, while two others^14^, ^15^ performed a comparison with groups with typical development and central auditory processing disorder; the others had only members with SLD and a control group. These differences provided variability in the findings and discussions, and only the results concerning the comparison of children with SLD to those with typical development were described here.

The studies indicate an increase in the latency of the BAEP-speech waves in the groups that had language alterations;^12^, ^14^, ^15^ Filippini et al.^16^ shows a delay only in the E wave, while Basu et al.^21^ made no observation regarding this aspect in the evaluation with speech stimulus. The articles indicate that there is a reduction in the amplitudes of all waves,^12^, ^15^, ^21^ showing that the increase in the presentation rate implies in a decrease in the amplitude of the responses;^21^ one of the studies,^16^ however, indicates no alterations in this parameter in the group with SLD. No changes were found regarding the VA complex area. The study by Filippini et al.,^16^ did not observe changes in the latency values of the BAEP-speech waves without competitive noise in the SLD group before and after the acoustically controlled auditory training.

It can be observed there is no consensus in the literature regarding the results related to the latency and amplitude of the BAEP-speech waves; considering that one of the studies indicates a decrease in amplitude by increasing the presentation rate, the importance of using the same stimulation parameters for the exam is verified, thus reducing the variability of the findings.

There is an agreement in the literature regarding the reduction of the frequency encoding phase blocking, especially at the higher ones, making it difficult to perceive frequency changes.^14^, ^15^, ^21^ The response is also deteriorated by the increase in the stimulus presentation rate,^21^ indicating perception deficits in the rapid transition of temporal-spectrum elements in the speech of children with SLD.

As for spectral data, one of the studies^21^ indicates that the peaks appear unchanged only when there are lower rates of presentation, with a reduction in amplitude when this characteristic of the acoustic stimulus is increased, whereas two other articles^14^, ^15^ did not observe differences in this regard between the groups with and without SLD. Studies agree that there is neural instability and reduced synchrony of speech responses in this population. Thus, it is possible to verify that children with SLD have difficulties in the subcortical and cortical encoding of speech sounds, impairing language development.

### Autism spectrum disorder

Of the studies that investigated individuals with ASD, only three^9^, ^11^, ^18^ presented numerical analyses of the results of the BAEP-speech. Ramezani et al.^11^ pointed out that there are higher latencies in all analyzed waves, but there are no differences in amplitude between the study and control groups, while Russo et al.^18^ identified only delays in components V, A, D and F and reduction in the F wave amplitude. Regarding the VA complex, the first study observed an increase in latency, with reduced duration and slope and, in contrast, the second study identified a prolonged duration of this component. The small number of studies that showed analyses of the characteristics of the BAEP-speech waves does not favor the possibility of consensus regarding the pathology findings with the examination performance.

One of the studies^9^ performed an assessment with BAEP-speech in two moments (T1 and T2) to observe the development of speech responses in the population with ASD. The children in the study group had lower V-wave latency and higher amplitudes of waves A and C in T2 compared to T1. When compared to individuals with typical development, the population with ASD had prolonged wave V and A latencies in T1, in addition to reduced wave E amplitude and F wave peak latency in T2, indicating development of auditory processing at the immature subcortical level in preschoolers with ASD.

The study carried out by Russo et al.,^22^ in contrast to the others, observed the pitch encoding in children with ASD, and identified a deficient encoding for speech pitch and less accurate tone tracking, highlighting a greater error in the speech frequency differentiation in individuals with ASD. Otto-Meyer et al.^20^ observed the stability of the responses in this population, evidencing the variability in response stability even in high-performance autists. The authors agree that in the same study group there are participants who have difficulty in the auditory processing of complex sounds, whereas others have similar responses to the group of children with typical development.

Two studies^11^, ^18^ indicated that there is less representation of the speech start being presented, with a prolonged initial duration of the response. Other studies^18^, ^22^ indicate the presence of phase block reduction, being presented with less accuracy. All the studies carried out on this topic agree there is a neural stability deficit, pointing out^20^, ^22^ the possibility that these individuals have receptive and temporal prosodic speech deficits. Such aspects may be related to the difficulty in the social use of communication, considering that the perception of prosodic speech elements and the ability to use them in language expression is an important aspect for communicational effectiveness.

### Phonological disorders

The two articles that investigated auditory processing for speech sounds in the population with Phonological Disorders (PD) differed on the assessment findings. Regarding latency, Ferreira et al.^10^ identified an increase in the values of all waves, however with significance only for the V, A, C, F and O waves, while Gonçalves et al.^17^ showed a delay only in the V and A components. Both indicate greater loss of acoustic cues in the transient portion of the stimulus. Only the last study mentioned analyzed wave amplitudes; however, there was no difference in the values between the study and control groups.

Regarding the characteristics of the VA complex, one of the studies^17^ indicated that there were no differences between the groups; in contrast, the second study^10^ identified a reduction in the wave slope and an increase in the area values, since they are inversely proportional measures, which may be indicative of an implicit reduction in neural activity during the appearance of the analyzed potential.

Both studies indicate that there are deficits in the perception of the temporal properties of speech sounds, which are related to changes in the synchronicity of neural generators that can impair the cortical processing of acoustic information. One of the studies^10^ indicates that the basis of the PD lies in the speech sound processing by the auditory system; thus, individuals with this alteration have neural encoding impairment of complex sounds. Studies agree that these difficulties can compromise language development skills and interfere with the individual’s social communication.

### Persistent developmental stuttering

The application of BAEP-speech in the population with persistent developmental stuttering was identified only in the study of Mozaffarilegha et al.,^19^ which used graphic visibility and fractality to observe the complexity of the responses to the exam in individuals with oral language pathology and with typical development.

The study group showed greater complexity in the graphic index, compared to the group without alterations. They observed that the visibility of the BAEP-speech graph shows an association between topology and fractality with the long-term memory of the auditory system in the brainstem in children with persistent developmental stuttering.

### Attention deficit/hyperactivity disorder

The literature search showed only one article that studied the application of the BAEP-speech in children with ADHD. Jafari et al.^13^ points out the difficulty in observing the presence of C and O waves in the study and control groups, whereas the other waves are easily verified.

The study indicates there are higher latencies of waves A, D, E, F and O, as well as an increase in the duration of the VA complex in children with ADHD. It also observed a prolongation in the beginning and displacement phases of the stimuli, corresponding to waves A and O, thus identifying that there is less synchronization of the neural responses at the beginning and at the end of the speech acoustic event in the children of the study group. They also showed delays in waves D, E and F, indicating greater difficulty in understanding the sustained elements of speech. As for the VA complex, the study indicated, in addition to the prolongation of duration, smaller amplitude and slope of the component, showing a reduction in the neural synchrony in children with ADHD.

A lower signal-to-noise ratio was also found, dividing the average pre-response amplitude by the measurement after stimulation, and the high physiological noise may have influenced the processing of the stimuli and the obtained results. Finally, they suggest there may be an interference of the afferent (bottom-up) and efferent (top-down) neural pathways in the processing of speech and non-speech stimuli in the population with ADHD.

## Conclusion

Children with language disorders showed different responses when performing the BAEP-speech when compared to children without language disorders. Among the main findings are delayed latencies, reduced amplitude, reduced phase block and changes in wave morphology in different pathologies. Due to the variation in the methodology, the parameters used in the stimulation and the assessed pathologies, it is not possible to generalize the findings of the studies.

## Funding

This work was carried out with the support of the Coordination for the Improvement of Higher Education Personnel – (CAPES) Brazil – Finance Code 001.

## Conflicts of interest

The authors declare no conflicts of interest.
